# Reproductive factors and risk of cardiovascular diseases and all-cause and cardiovascular mortality in American women: NHANES 2003–2018

**DOI:** 10.1186/s12905-024-03055-6

**Published:** 2024-04-05

**Authors:** Yufeng Yan, Hongjing Lu, Song Lin, Yaguo Zheng

**Affiliations:** Department of Cardiology, Nanjing First Hospital, Nanjing Medical University, No. 68 Changle road, Qinhuai District, Nanjing, Jiangsu 210008 China

**Keywords:** Reproductive factors, Cardiovascular diseases, Nhanes, Cardiovascular and all-cause death, Women’s health

## Abstract

**Background:**

The evidence regarding the association of reproductive factors with cardiovascular diseases (CVDs) is limited.

**Aims:**

To investigate the relationship of reproductive factors with the risk of CVDs, as well as all-cause and cardiovascular mortality.

**Methods:**

This study included 16,404 adults with reproductive factors from the National Health and Nutrition Examination Survey (NHANES) and followed up until 31 December 2019. Logistic models and restricted cubic spline models were used to assess the association of reproductive factors with CVDs. COX proportional hazards models and restricted cubic spline models, with adjustment for potential confounding, were employed to analyze the relation between reproductive factors and cardiovascular and all-cause death.

**Results:**

There is a nonlinear relationship between age at menarche and CVDs. Age at menarche ≤ 11(OR 1.36, 95% CI 1.10–1.69) was associated with an increased risk of CVDs compared to ages 12–13 years. Age at Menopause ≤ 44 (OR 1.69, 95% CI 1.40–2.03) was associated with increased CVDs compared to age 35–49 years. Number of pregnancies ≥ 5(OR 1.26, 95% CI 1.02–1.55) was associated with an increased risk of CVDs compared to one pregnancy. In continuous variable COX regression models, a later age at menopause (HR 0.98, 95% CI 0.97–0.99) and a longer reproductive lifespan (HR 0.98, 95% CI 0.97–0.99) were associated with a decreased risk of all-cause death. A later age at menopause (HR 0.98, 95% CI 0.97–0.99) and a longer reproductive lifespan (HR 0.98, 95% CI 0.97–0.99) were associated with a decreased risk of cardiac death.

**Conclusions:**

Female reproductive factors are significant risk factors for CVDs American women.

**Supplementary Information:**

The online version contains supplementary material available at 10.1186/s12905-024-03055-6.

## Introduction

Cardiovascular diseases(CVDs) are among the most serious diseases that harm health and cause death globally [1]. Through interventions targeting high-risk factors, the incidence of CVDs has been effectively managed. Although the mortality and morbidity rates of CVDs have decreased, the CVD burden has increased in women [2]. Sex has a profound impact on CVDs [3]. Before menopause, women generally exhibit a lower risk of CVDs than men. Changes in female sex hormones occur throughout the reproductive process, from menarche to pregnancy, childbirth, and menopause. These hormone levels affect a woman’s blood lipid levels, blood pressure, blood sugar, and inflammation, subsequently influencing cardiovascular health [4]. Reproductive health in women plays a vital role in the physiological and pathological processes of the cardiovascular system. Therefore, evaluating the influence of reproductive factors on CVDs is crucial to reducing their incidence in women.

By analyzing the impact of these factors on all-cause mortality and cardiovascular mortality, we can gain a deeper understanding of their effects on women’s overall health. Throughout a woman’s life, various reproductive factors play a significant role, encompassing the entire process from female reproductive system development to senescence. Some research has demonstrated a correlation between early menarche and an increased risk of all-cause mortality or cardiovascular mortality [5–8]. However, contrasting findings from studies on European populations suggest that late menarche is associated with an elevated risk of cardiovascular death, while early menarche does not show the same association [9]. Additionally, some studies have reported an association between an earlier age of menopause and higher mortality rates, particularly in relation to CVDs [10]. Nevertheless, the relationship between female reproductive factors and the risk of all-cause and cardiovascular mortality death in women remains controversial.

**Fig. 1:**
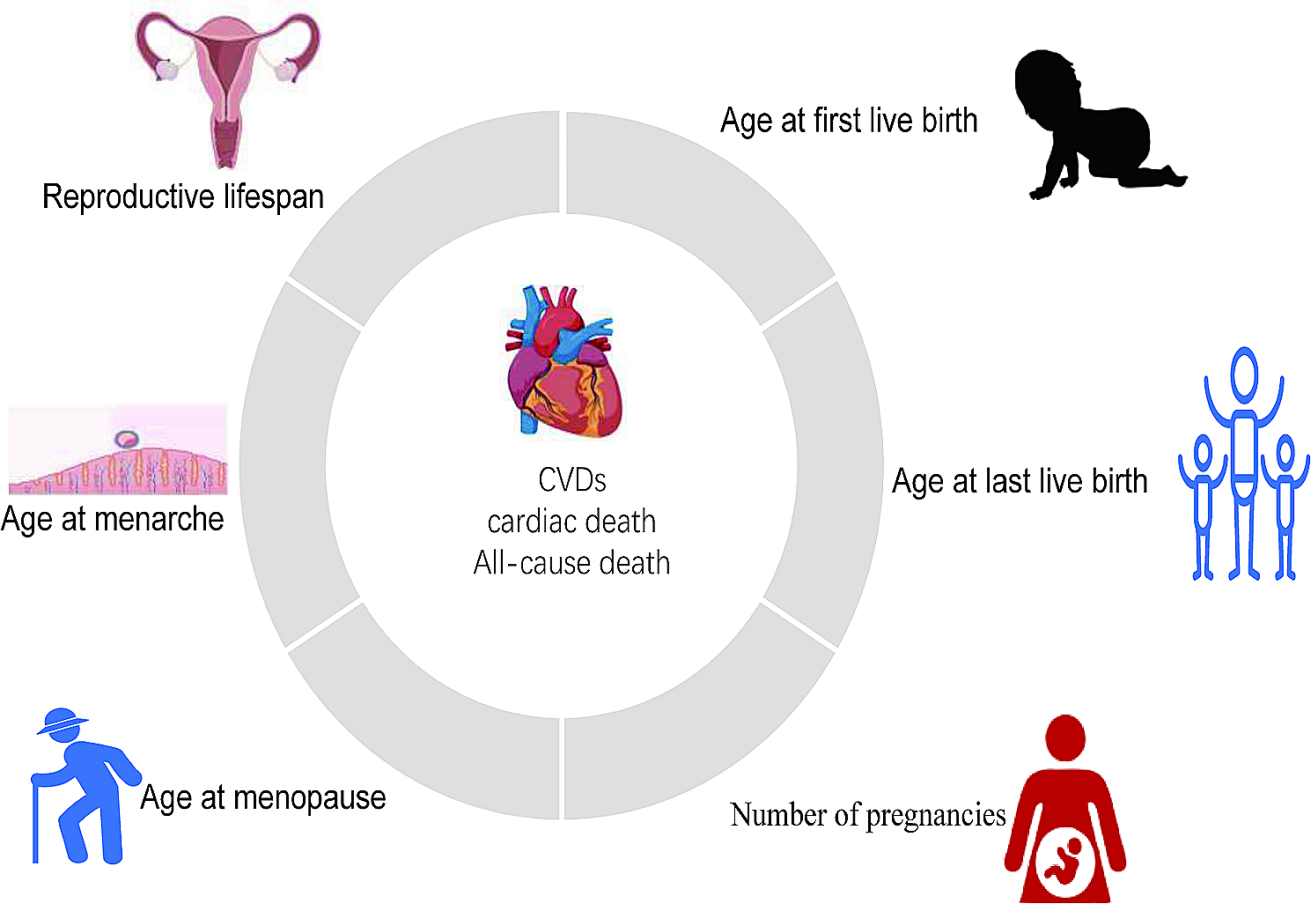
Highlight

The development of treatment guidelines for CVDs has predominantly focused on male patients, neglecting the importance of studying how female reproductive factors contribute to cardiovascular health. This study aimed to analyze the National Health and Nutrition Examination Survey (NHANES) database to explore the relationship between reproductive factors(such as age at menarche, age at menopause, reproductive span, age at first live birth, and number of live births) and CVDs, all-cause death, and cardiovascular death in women. Highlight in our research was showed in Fig. 1. The findings from this study offer valuable insights for the early identification and management of female-specific cardiovascular risk factors.

## Methods

### Study population

We downloaded data from the 2003–2018 NHANES to explore the associations between reproductive factors and CVDs. The cohort study sample comprised individuals who participated in the NHANES survey from 2003 to 2018, a population-based survey designed to collect health and nutritional statistics of adults and children in the United States [11]. The NHANES is a nationally conducted survey encompassing a range of dimensions, including in-person household interviews that address demographic, socioeconomic, dietary, and health-related inquiries, health screenings involving medical data, and the collection of anthropometric and laboratory measurements at designated mobile screening centers (MECs) [12]. The NHANES protocol underwent revision and received endorsement from the NCHS Ethics Review Committee, with written informed consent obtained from all participants [13]. It uses a multi-stage sampling design that can represent different age, race, gender, and geographic distributions of the US population, providing extensive data that reflects the real population characteristics. A total of 28,063 participants included in the NHANES from 2003 to 2014. Of these, participants had mortality follow-up data available through December 31, 2019 from the US National Death Index(NDI). We excluded participants for a lack of information, 7237 for a lack of Information on reproductive factors, 1696 for a lack of poverty income ratio,12 for a lack of education information,230 for a lack of BMI,830 for a lack of DM, 562 for a lack of blood pressure, and 1092for a lack of CVDs. We finally included 16,404 participants in the final analysis.

### Outcome

Outcome were obtained from the National Death Index through 31 December 2019 to identify mortality status. The primary cause of death was determined according to ICD-10 codes. Outcomes were be defined as all-cause and cardiovascular mortality. Cardiovascular mortality was defined using ICD-10 codes 100–109, 111, 113, 120-I51.

CVDs include conditions such as coronary heart disease, congestive heart failure, heart attack, stroke, and angina.

### Study covariates

Demographic information (sex, age, family income-poverty ratio, race/ethnicity and educational level) and lifestyles(smoking, drinking alcohol) were collected during in-home interviews. Body mass index (BMI) was measured at mobile examination centers. BMI is calculated by dividing an individual’s weight by the square of their height. Race/ethnicity was categorized as Mexican American, non-Hispanic white, non-Hispanic black, other Hispanic, and others. Education levels were categorized as follows: less than 9th grade, 9-11th grade (includes 12th grade with no diploma), high school graduate/GED or equivalent, some college or AA degree, and college graduate or above. Hypertension was defined as either a self-reported medical history or a blood pressure reading of 130/80 mmHg or higher. Diabetes was determined through a combination of self-reported medical history, glycosylated hemoglobin A1c level of 6.5% or higher, or fasting plasma glucose level of 126 mg/dL or higher.

### Statistical analyses

Sampling weights were considered in our study due to the complex sampling design of the NHANES. Means ± standard deviation are used for continuous variables, and percentages are utilized for categorical variables. We used a logistic model to assess the correlation between reproductive factors and CVDs events. The logistic model assumes that the relationship between the predictor variables and the log odds of the binary response variable is linear. A common limitation of logistic models is that assumptions about the linear relationship between the independent and response variables may not be realistic. In addition, logistic models suffer from problems of covariance, overfitting and sample imbalance. To enhance the robustness of the logic model, measures such as introducing regularization terms to control the complexity of the model or using cross-validation methods to assess the performance of the model can be taken [14]. Multivariable Cox proportional hazards models were used to assess the association of reproductive factors with cardiovascular death and all-cause death. All reproductive factors were assessed as both continuous and categorical variables.

The COX proportional hazards model is a statistical method used for survival analysis that assumes that the hazard function varies proportionally. The hazard function describes the probability of an event occurring at a given point in time. The Cox proportional risk model assumes that the effects of risk factors are linear and may not capture the effects of non-linear relationships. To enhance the robustness of the COX proportional hazards model, some sensitivity analyses can be performed [15].The non-linear relationship between reproductive factors and cardiovascular events, all-cause death, and cardiovascular death was evaluated using restricted cube plots (4 knots). The relationship between the predictor variable and the response variable is assumed to be smooth and can be represented by restricted cubic splines. A limitation of the restricted cubic spline model is the need to select the appropriate number and location of nodes. Wrong selection may lead to overfitting or underfitting problems. In addition, the restricted cubic spline model may not be robust enough for regions where there is a lack of data or sparse data. To enhance the robustness of the restricted cubic spline model, cross-validation or information criteria (e.g., AIC, BIC) can be used to help select the optimal number and location of nodes. In addition, for rare events or small amounts of data, consider using alternative non-parametric regression methods [16].Categorical variables were used to evaluate the relationship between reproductive factors and cardiovascular events, all-cause death, and cardiovascular death. Two models were used to analyze confounding factors. Model 1 makes adjustments for age. Model 2 was adjusted for race, BMI, alcohol consumption, smoking status, hypertension, diabetes mellitus, and education, Statistical analyses were conducted using R software version 4.1.0, P-value < 0.05 was considered statistically significant.

### Sensitivity analysis

To enhance the robustness of our findings, we performed a sensitivity analysis. We performed repeated analyses in diabetic, non-diabetic, hypertensive, and non-hypertensive populations.

## Results

### Baseline characteristics of the study population

The baseline information is presented in Table 1. During a median follow-up of 105(IQR: 59–148) months, 1693 participants died, and 459 individuals experienced cardiovascular mortality.1572 participants was recorded as having CVDs.

**Table 1:** Baseline characteristics of the study population

Variable	Total
At age Menarche, y	12.70 ± 0.02
Age, y	48.22 ± 0.25
Poverty-income ratio	2.95 ± 0.03
Body mass index, kg/m2	29.09 ± 0.10
Alcohol consumption	
Missing	12102175.08(13.37)
No	24670017.57(27.26)
Yes	53719548.35(59.36)
Race	
Mexican American	6419253.04( 7.09)
Non-Hispanic Black	10197540.29(11.27)
Non-Hispanic White	63250421.66(69.90)
Other Hispanic	4484634.05( 4.96)
Other Race - Including Multi-Racial	6139891.94( 6.79)
Education	
9-11th grade (Includes 12th grade with no diploma)	9008261.61( 9.95)
College graduate or above	26411609.41(29.19)
High school graduate/GED or equivalent	20523026.66(22.68)
Less than 9th grade	4045007.76( 4.47)
Some college or AA degree	30503835.55(33.71)
Smoke	
Former	19206204.48(21.22)
Missing	31429.73( 0.03)
Never	54391361.42(60.11)
Now	16862745.36(18.63)
Diabetes Mellitus	
DM	11857004.74(13.10)
IFG	3060212.57( 3.38)
IGT	2989888.69( 3.30)
No	72584634.99(80.21)
Hypertension	
No	56544122.58(62.49)
Yes	33947618.41(37.51)
CVD	
No	83449764.35(92.22)
Yes	7041976.64( 7.78)

### Incidence of CVD according to reproductive factors

**Fig. 2:**
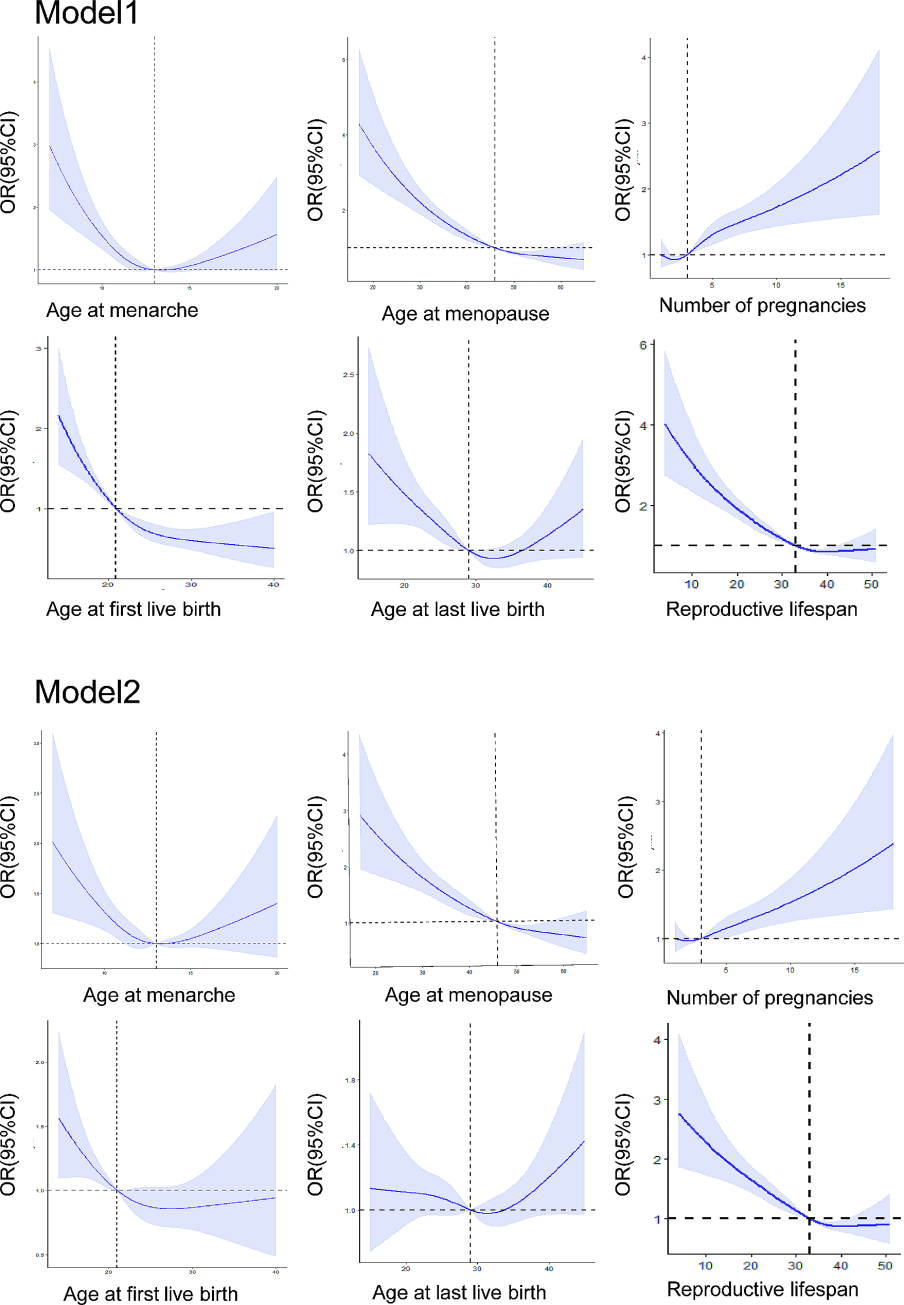
Effect plots for nonlinear associations of reproductive factors with incident CVDs

The reproductive factors showed a relationship with CVDs in adjusted models. A nonlinear relationship was observed between age at menarche with CVDs, as indicated in the cube plot (Fig. 2). Linear relationships were established between age at menopause, reproductive lifespan, and number of pregnancies with CVDs in the multivariable model. In continuous variable logistic regression models, a later age at menopause (OR 0.96, 95% CI 0.96–0.97), a longer reproductive life span(OR 0.97, 95% CI 0.96–0.97),and a greater number of pregnancies(OR 1.06,95% CI 1.02–1.09) were associated with a decreased risk of CVDs(Table 2). A greater number of pregnancies (OR 1.06, 95% CI 1.02–1.09) was associated with an increased risk of CVDs. After categorizing the variables, in the multivariate model(model 2), an age at menarche ≤ 11 was associated with increased CVDs, while ages 14–15 and ≥ 16 were not significantly associated with increased CVDs compared to the reference. Age at menopause ≤ 44(OR 1.69, 95% CI 1.40–2.03) was associated with increased CVDs, whereas ages 50–54 and ≥ 55 were not. A reproductive lifespan of ≤ 32 was associated with increased CVDs, while spans of 36–38, 39–41, and ≥ 42 were not. Maternal age at first live birth of ≤ 19, 24–26, and ≥ 27 and maternal age at last live birth of ≤ 26, 30–34, 35–39, and ≥ 40 were not associated with increased CVDs compared to the reference. A number of pregnancies ≥ 5(OR 1.26, 95% CI 1.02–1.55) was associated with an increase in CVDs, whereas numbers 1,3,4 were not.

**Table 2:** Assocaition of reproductive factors with incident CVDs

Reproductive Factors	Age-Adjusted Model		Multivariable Model	
	OR (95% CI)	*P* Value	OR (95% CI)	*P* Value
≤ 11	1.55(0.03–1.89)	0.000	1.36(1.10–1.69)	0.005
12–13	ref			
14–15	1.07(0.16–1.27)	0.447	1.06(0.89–1.27)	0.497
≥ 16	1.35(0.10–1.71)	0.016	1.26(1.00-1.59)	0.054
Age at menopause				
≤ 44	1.84(0.90–2.22)	0.000	1.69(1.40–2.03)	0.000
35–49	ref			
50–54	0.83(0.87–1.04)	0.102	0.93(0.73–1.18)	0.538
≥ 55	0.87(0.11–1.14)	0.316	0.93(0.71–1.22)	0.600
Reproductive lifespan				
≤ 32	1.79(0.08–2.21)	0.000	1.63(1.33-2.00)	0.000
33–35	ref			
36–38	0.92(0.79–1.21)	0.565	0.94(0.70–1.26)	0.660
39–41	0.80(0.03–1.10)	0.174	0.90(0.65–1.26)	0.540
≥ 42	0.97(0.11–1.26)	0.814	1.02(0.77–1.35)	0.904
Maternal age at first live birth				
≤ 19	1.37(0.23–1.72)	0.006	1.04(0.81–1.33)	0.742
21–23	ref			
24–26	0.67(0.97 − 0.94)	0.021	0.77(0.53–1.11)	0.159
≥ 27	0.50(0.11–0.69)	0.000	0.75(0.52–1.08)	0.123
Maternal age at last live birth				
≤ 26	1.26(0.98–1.62)	0.066	1.08(0.85–1.37)	0.545
27–29	ref			
30–34	0.90(0.70–1.16)	0.427	1.00(0.77–1.30)	0.979
35–39	0.95(0.72–1.26)	0.716	1.09(0.82–1.44)	0.561
≥ 40	1.39(0.87–2.22)	0.166	1.44(0.89–2.32)	0.135
Number of pregnancies				
1	0.96(0.03–1.28)	0.793	1.00(0.74–1.34)	0.984
2	ref			
3	0.89(0.17–1.13)	0.328	0.92(0.71–1.20)	0.551
4	1.21(0.21–1.58)	0.165	1.13(0.85–1.50)	0.393
≥ 5	1.52(0.10–1.84)	0.000	1.26(1.02–1.55)	0.034
Age at menarche	0.95(0.10–0.99)	0.017	0.95(0.91–1.01)	0.105
Age at menopause	0.95(0.11–0.96)	0.000	0.96(0.96–0.97)	0.000
Reproductive lifespan	0.96(0.11–0.96)	0.957	0.97(0.96–0.97)	0.000
Maternal age at first live birth	0.92(0.11–0.95)	0.000	0.97(0.95-1.00)	0.057
Maternal age at last live birth	0.99(0.11-1.00)	0.105	1.01(0.99–1.03)	0.520
Number of pregnancies	1.10(0.10–1.13)	0.000	1.06(1.02–1.09)	0.001

### Cardiac death according to reproductive factors

**Fig. 3:**
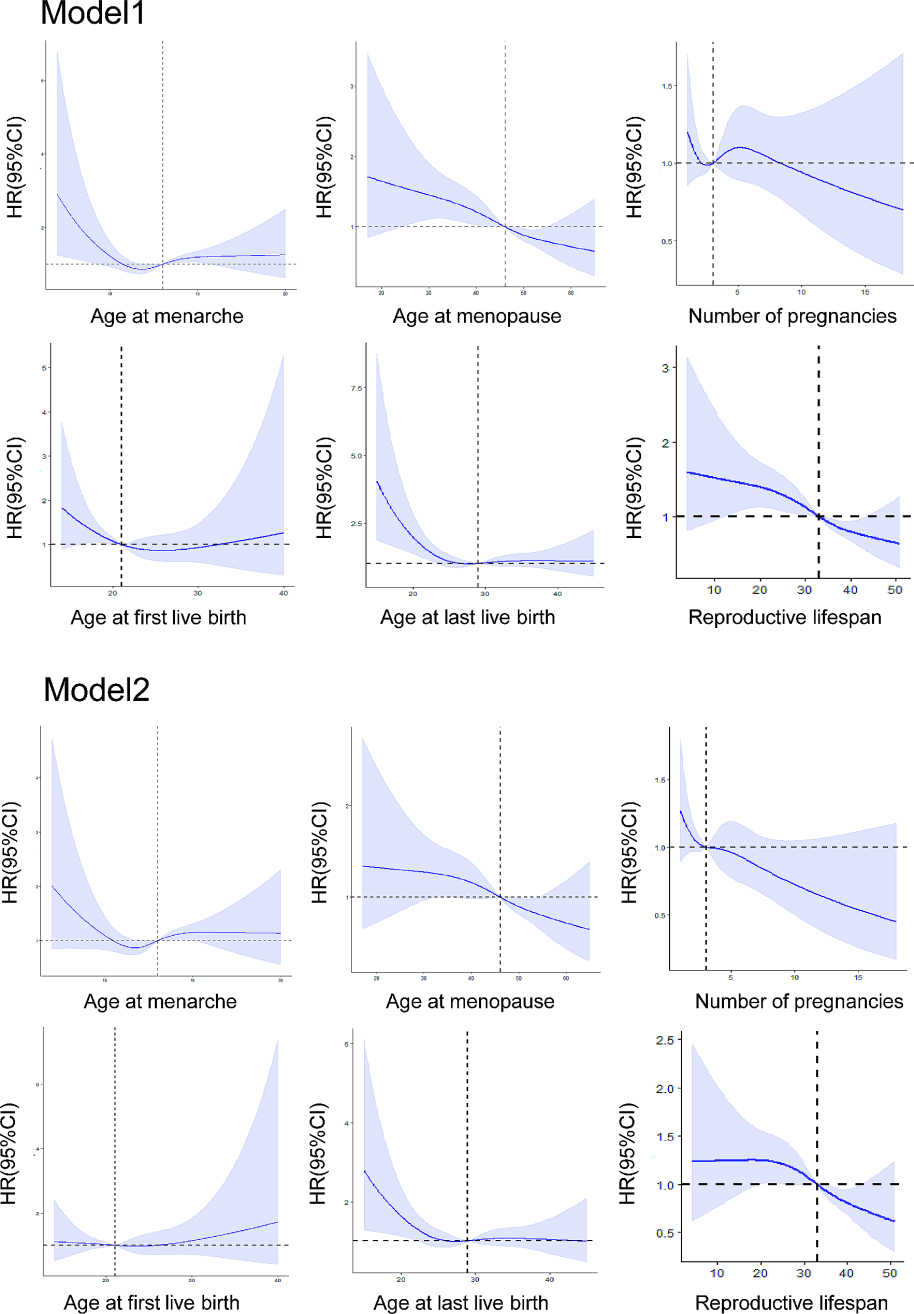
Effect plots for nonlinear associations of reproductive factors with incident cardiac death

A non-linear relationship between reproductive factors and cardiovascular death was indicated in the cube plot (Fig. 3). Linear relationships were observed between age at menopause, reproductive lifespan, and number of pregnancies with cardiac death in the multivariable model. In continuous variable COX regression models, a later age at menopause (HR 0.98, 95% CI 0.97–0.99), a longer reproductive lifespan (HR 0.98, 95% CI 0.97–0.99) were associated with a decreased risk of cardiac death (Table 3). After categorizing the variables, in the multivariate model(model 2), ages at menarche of ≤ 11, 14–15, and ≥ 16 were not significantly associated with increased cardiovascular death compared to the reference. Ages at menopause of ≤ 44, 50–54, and ≥ 55 were not associated with increased risk. A reproductive lifespan of ≤ 32 (HR 1.62, 95% CI 1.10–2.39) was associated with increased risk, while spans of 36–38, 39–41, and ≥ 42 were not. Maternal age at first live birth of ≤ 19(HR 1.63, 95% CI 1.10–2.41) was associated with increased cardiovascular death risk, while ages 24–26 and ≥ 27 were not. Maternal ages at last live birth of ≤ 26, 30–34, 35–39, and ≥ 40 and number of pregnancies of ≥ 5, 1, 3, 4 were not associated with increased risk compared to the reference.

**Table 3:** Assocaition of reproductive factors with incident cardic death

Reproductive Factors	Age-Adjusted Model		Multivariable Model	
	HR (95% CI)	*P* Value	HR (95% CI)	*P* Value
≤ 11	1.06(0.78–1.44)	0.696	0.97(0.71–1.31)	0.828
12–13	ref			
14–15	1.11(0.82–1.51)	0.488	1.00(0.75–1.34)	0.989
≥ 16	1.31(0.91–1.88)	0.141	1.14(0.81–1.60)	0.463
Age at menopause				
≤ 44	1.34(0.96–1.87)	0.085	1.21(0.87–1.68)	0.256
35–49	ref			
50–54	0.91(0.67–1.24)	0.562	0.94(0.68–1.28)	0.677
≥ 55	0.80(0.54–1.18)	0.260	0.81(0.53–1.22)	0.309
Reproductive lifespan				
≤ 32	1.73(1.21–2.49)	0.003	1.62(1.10–2.39)	0.014
33–35	ref			
36–38	1.18(0.83–1.69)	0.356	1.19(0.81–1.76)	0.367
39–41	0.97(0.65–1.45)	0.886	1.07(0.71–1.63)	0.739
≥ 42	0.89(0.56–1.41)	0.614	0.91(0.55–1.51)	0.719
Maternal age at first live birth				
≤ 19	2.19(1.55–3.09)	0.000	1.63(1.10–2.41)	0.015
21–23	ref			
24–26	1.30(0.80–2.12)	0.294	1.45(1.37–2.46)	0.172
≥ 27	1.15(0.69–1.90)	0.595	1.36(1.18–2.44)	0.309
Maternal age at last live birth				
≤ 26	1.21(0.69–2.10)	0.504	1.11(0.64–1.92)	0.709
27–29	ref			
30–34	1.09(0.60–2.01)	0.774	1.10(0.58–2.07)	0.776
35–39	1.12(0.62-2.00)	0.714	1.09(0.61–1.96)	0.764
≥ 40	1.31(0.77–2.21)	0.315	1.06(0.63–1.78)	0.838
Number of fetation				
1	1.44(0.89–2.32)	0.134	1.60(0.97–2.63)	0.067
2	ref			
3	1.09(0.75–1.61)	0.644	1.19(0.80–1.76)	0.398
4	1.41(0.94–2.12)	0.096	1.35(0.89–2.05)	0.155
≥ 5	1.40(1.00-1.96)	0.052	1.08(0.75–1.55)	0.666
Age at menarche	1.05(0.98–1.12)	0.188	1.04(0.97–1.11)	0.279
Age at menopause	0.97(0.96–0.99)	0.000	0.98(0.97–0.99)	0.001
Reproductive lifespan	0.97(0.96–0.98)	0.000	0.98(0.97–0.99)	0.000
Maternal age at first live birth	0.95(0.91-1.00)	0.031	1.00(0.95–1.05)	0.976
Maternal age at last live birth	0.98(0.95–1.02)	0.363	0.99(0.96–1.02)	0.470
Number of pregnancies	1.02(0.98–1.07)	0.328	0.96(0.91–1.02)	0.209

### All-cause mortality according to reproductive factors

**Fig. 4:**
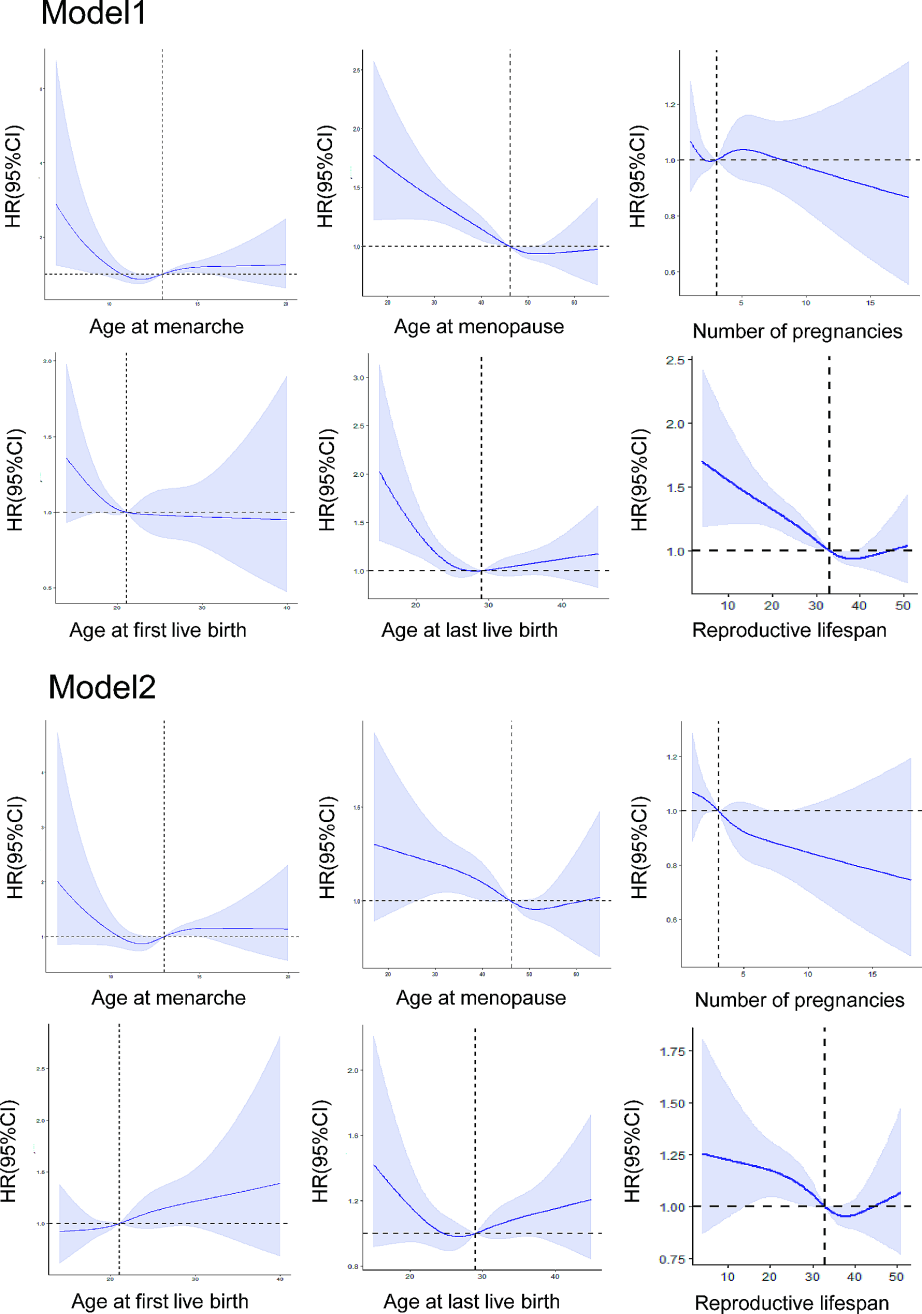
Effect plots for nonlinear associations of reproductive factors with incident all-cause mortality

A non-linear relationship between reproductive factors and all-cause death was indicated in the cubic plot (Fig. 4). A linear relationship were established between age at menopause, reproductive lifespan, and number of pregnancies with cardiac death in the multivariable model. In continuous variable COX regression models, a later age at menopause (HR 0.98, 95% CI 0.97–0.99), a longer reproductive lifespan (HR 0.98, 95% CI 0.97–0.99) were associated with a decreased risk of all-cause death (Table 4). After categorizing the variables, in the multivariate model(model 2), ages at menarche of ≤ 11, 14–15, and ≥ 16 were not significantly associated with increased all-cause death compared to the reference. Ages at menopause of ≤ 44, 50–54, and ≥ 55 were not associated with increased risk. The reproductive lifespans of ≤ 32, 36–38, 39–41, and ≥ 42 were not associated with increased risk compared to the reference. Maternal age at first live birth of 24–26(HR 1.37, 95% CI 1.06–1.76) was associated with increased all-cause death, while ages ≤ 19 and ≥ 27 were not. Maternal ages at last live birth of ≤ 26, 30–34, 35–39, and ≥ 40 and numbers of pregnancies of ≥ 5, 1, 3, 4 were not associated with increased all-cause death compared to the reference.

**Table 4:** Assocaition of reproductive factors with incident death

Reproductive Factors	Age-Adjusted Model		Multivariable Model	
	HR (95% CI)	*P* Value	HR (95% CI)	*P* Value
≤ 11	1.13(0.97–1.31)	0.116	1.05(0.91–1.22)	0.477
12–13	ref			
14–15	1.03(0.88–1.21)	0.731	0.95(0.81–1.12)	0.562
≥ 16	1.16(0.92–1.46)	0.209	1.05(0.83–1.32)	0.687
Age at menopause				
≤ 44	1.22(1.03–1.44)	0.021	1.12(0.94–1.34)	0.191
35–49	ref			
50–54	0.90(0.76–1.07)	0.247	0.96(0.81–1.15)	0.663
≥ 55	0.87(0.70–1.09)	0.235	0.93(0.72–1.19)	0.542
Reproductive lifespan				
≤ 32	1.32(1.07–1.63)	0.010	1.24(0.99–1.57)	0.065
33–35	ref			
36–38	1.08(0.84–1.39)	0.525	1.14(0.86–1.51)	0.350
39–41	0.79(0.62–1.02)	0.073	0.88(0.67–1.16)	0.354
≥ 42	1.03(0.80–1.32)	0.840	1.10(0.83–1.46)	0.494
Maternal age at first live birth				
≤ 19	1.44(1.17–1.77)	0.000	1.10(0.87–1.39)	0.428
21–23	ref			
24–26	1.23(0.96–1.57)	0.100	1.37(1.06–1.76)	0.015
≥ 27	0.97(0.73–1.29)	0.840	1.18(0.85–1.62)	0.316
Maternal age at last live birth				
≤ 26	1.09(0.87–1.36)	0.462	0.98(0.80–1.22)	0.890
27–29	ref			
30–34	0.95(0.73–1.23)	0.703	0.96(0.73–1.26)	0.754
35–39	1.22(0.92–1.63)	0.163	1.30(0.99–1.72)	0.061
≥ 40	1.33(0.92–1.91)	0.131	1.18(0.81–1.73)	0.380
Number of pregnancies				
1	1.11(0.89–1.39)	0.354	1.16(0.93–1.45)	0.190
2	ref			
3	1.03(0.85–1.27)	0.741	1.12(0.91–1.38)	0.271
4	1.17(0.96–1.44)	0.129	1.13(0.92–1.40)	0.242
≥ 5	1.21(1.04–1.42)	0.017	1.00(0.84–1.19)	0.990
Age at menarche	1.00(0.96–1.04)	0.976	0.99(0.96–1.03)	0.672
Age at menopause	0.98(0.97–0.99)	0.000	0.99(0.98-1.00)	0.006
Reproductive lifespan	0.98(0.98–0.99)	0.000	0.99(0.98-1.00)	0.016
Maternal age at first live birth	0.98(0.95-1.00)	0.021	1.02(1.00-1.04)	0.118
Maternal age at last live birth	1.00(0.98–1.01)	0.966	1.01(0.99–1.02)	0.290
Number of pregnancies	1.02(0.99–1.05)	0.160	0.98(0.95–1.01)	0.145

### Sensitivity analyses

In all sensitivity analyses, most of the results remained stable and consistent. The sensitivity analysis results are shown in (Supplementary materials table S1S2 and S3).

## Discussion

This is the first comprehensive assessment of the association between female reproductive factors and all-cause mortality, cardiogenic death, and all-cause death using the NHANES database. We found that, after controlling for known CVDs risk factors, a later age at menopause, a longer reproductive lifespan, and fewer pregnancies were associated with a significantly lower risk of CVDs. Our study also found that a shorter reproductive lifespan and earlier age at menopause were associated with an increased risk of both all-cause and cardiovascular death.

We found that there is a nonlinear relationship between age at menarche and CVDs in our study. Previous studies have identified various patterns of association between age at menarche and CVDs risk in women. Some studies have shown an increased risk of CVDs with early age at menarche [17], while others have indicated an increased risk with later age at menarche [18]. In a retrospective study, earlier age at menarche was associated with a higher risk of heart failure in women [19]. Some studies have suggested that early menarche is associated with an increased risk of CVDs in women [20–22]. However, a study from Korea determined that late menarche was associated with an increased risk of MI, but early age at menarche was not [18]. Another study of 1,088,992 premenopausal women found that late menarche was associated with an increased risk of all-cause disease, while early menarche was not [23]. Both later and earlier menarche are influenced by female adolescent BMI, hormones, and renal hormones. Beyond the relationship between age at menarche (AAM) and CVDs, we also explored its association with all-cause mortality. We found no significant relationship between AAM and all-cause mortality. Yet, some researchers have suggested that AAM is linked to all-cause mortality [20]. Additionally, a recent systematic review showed an inverse association between age at menarche and both all-cause mortality and mortality due to ischemic heart disease [24].

Our study confirms that early age at menopause significantly increases the risk of all-cause diseases. This conclusion is consistent with numerous other studies that have found premature menopause, whether natural or surgical, to be a risk factor for various all-cause diseases [25]. The decrease in endogenous estrogen secretion in postmenopausal women significantly affects low-density lipoprotein control, which may explain why postmenopausal women have a lower incidence of coronary heart disease compared to men [26]. In our study, even after adjusting for common risk factors, premature menopause was associated with a heightened risk of all-cause diseases. By identifying women with premature menopause, targeted prevention could reduce the incidence of all-cause diseases. Our research also determined that a shorter reproductive lifespan was linked to increased all-cause risk and cardiac death. This might result from the combined effects of irregular estrogen production timing and insufficient estrogen exposure. The Women’s Ischemia Syndrome Evaluation study noted a connection between disrupted ovulatory cycles characterized by hypoestrogenemia of hypothalamic origin and angiographic CAD (Coronary Artery Disease) [27].

We found no significant association between the first or last live birth and the occurrence of all-cause diseases (CVDs) or all-cause mortality. However, a study using data from the UK Biobank indicated that a younger maternal age at the time of the first or last live birth is a risk factor for heart failure (HF) events [19]. Previous studies have suggested that a younger maternal age at conception might be associated with CVD [28, 29]. A systematic review of observational studies from 1980 to 2016 found the relationship between early age at first live birth and all-cause disease risk to be uncertain, though there is some evidence suggesting a potential increase in risk. Of the studies reviewed, ten found that women with an early age at first live birth had a higher risk of developing CVDs, while two found that women with a later age of first birth had a greater risk of developing all-cause disease [30]. Studies have shown a U-shaped association between age at first birth and death from all-cause disease and all-cause mortality [28]. While our study did not replicate these findings, we did determine that an early age at first birth was associated with an increased risk of all-cause death.

The study identified a linear relationship between the number of pregnancies and the increased risk of all-cause disease. Some research has indicated that the higher the number of live births correlates with a greater risk of HF [19]. This relationship might be due to hormonal changes during pregnancy, increased cardiac workload, abnormal blood sugar levels, dyslipidemia, and the long-term effects of pregnancy on all-cause health. Additionally, multiple pregnancies can lead to future weight gain and the onset of metabolic syndrome [31]. In a large prospective cohort, researchers found that a higher number of pregnancies correlated with a greater risk of subsequent atrial fibrillation [32]. Repeated exposure to metabolic, physiological, and hormonal changes during pregnancy, combined with increased cardiac workload, might contribute to the onset of atrial fibrillation later in life. During pregnancy, there are notable changes in hormone levels, especially increases in estrogen and progesterone. These shifts can cause a metabolic slowdown, potentially leading to fat accumulation in the abdominal area [33]. Based on previous studies, it has been reported that early maternal age at first birth is associated with unfavorable cardiovascular risk profiles in both men and women [34]. In a recent meta-analysis, it was found that women who have ever given birth have an increased risk of cardiovascular disease (CVD), with a 4% increased risk for each birth. This association is likely attributed to metabolic changes that occur during pregnancy [35]. However, more research is needed to fully understand this connection.

Importantly, our study did not find a significant relationship between the number of pregnancies and all-cause mortality. Some research has identified a nonlinear relationship between number of pregnancies and all-cause death [36], while other studies have found no such connection [37]. The relationship between the number of pregnancies and mortality is intricate, influenced by various factors like chronic diseases, race, and socioeconomic status. More investigation is essential to decipher the specific mechanisms behind these associations and to analyze the effects of repeated pregnancies on long-term health outcomes. There are practical challenges in implementing the assessment of female reproductive factors in current practice. One potential approach could involve the development of standardized questionnaires or assessment tools that can be administered during routine medical appointments. These questionnaires could cover various reproductive factors such as age at menarche, age at menopause, number of pregnancies. In the future, reproductive factors in women with cardiovascular disease should be included in the evaluation of questionnaires to guide clinical practice.

### Study strengths and limitations

This study has several advantages over previous studies. The study benefits from an extended follow-up period, enabling a thorough assessment of the relationship between reproductive factors and all-cause disease, as well as all-cause mortality. This research examines a broad spectrum of reproductive factors, offering a comprehensive understanding of their effects on all-cause health outcomes. Utilizing the NHANES database ensures national representativeness, which enhances the generalizability of the results to other populations. The analysis considers the influence of race, allowing for a more precise evaluation of the relationships between reproductive factors and health outcomes across various racial groups. However, our study has some limitations. Firstly, relying on self-reported questionnaire data might lead to recall bias, as participants may not remember specific reproductive factors accurately, potentially affecting the study’s conclusions. Secondly, the study focuses on six specific reproductive factors, but it fails to consider other relevant aspects such as hormone use, breastfeeding, or a history of reproductive system surgeries. Exploring the effects of these factors on overall health outcomes is necessary and should be further investigated. Thirdly, as patient baseline data can change over time, this variability might introduce bias into the study’s findings. In addition, our study failed to provide a more detailed analysis of the specific causes of cardiovascular and all-cause mortality. Lastly, The population included in this study is the US population, and its applicability to other populations requires further investigation.

## Conclusion

In summary, we discovered a significant association between female reproductive factors and the risk of all-cause disease in American women. Age at menarche、 age at menopause and reproductive lifespan are independent risk factors for CVDs. Our findings emphasize the importance of including an evaluation of reproductive factors in the assessment of women’s overall health risk.

## Electronic supplementary material

Below is the link to the electronic supplementary material.

Supplementary Material 1

Supplementary Material 2

Supplementary Material 3

Supplementary Material 4

## Data Availability

Data for this study were sourced from National Health and Nutrition Examination Survey (NHANES) and available here: https://www.cdc.gov/nchs/nhanes/index.htm.
