# Correction: Reproductive factors and risk of cardiovascular diseases and all-cause and cardiovascular mortality in American women: NHANES 2003–2018

**DOI:** 10.1186/s12905-025-03609-2

**Published:** 2025-02-21

**Authors:** Yufeng Yan, Hongjing Lu, Song Lin, Yaguo Zheng

**Affiliations:** Department of Cardiology, Nanjing First Hospital, Nanjing Medical University, No. 68 Changle Road, Qinhuai District, Nanjing, Jiangsu 210008 China

**Correction: BMC Women's Health 24, 222 (2024)**

**https://doi.org/10.1186/s12905-024-03055-6**

In the sentence beginning ‘There is a nonlinear relationship… compared to ages 12–13 years.' in the Results heading under the Abstract section of this article [1], ‘Age at menopause’ should have been read correctly as ‘Age at menarche’.

The corrected sentence should read as ‘Age at menarche ≤ 11 (OR 1.36, 95% CI 1.10–1.69) was associated with an increased risk of CVDs compared to ages 12–13 years. Age at Menopause ≤ 44 (OR 1.69, 95% CI 1.40–2.03) was associated with increased CVDs compared to age 35–49 years’. The original article has been corrected.
