# Supplementary material for: Reproductive factors and risk of cardiovascular diseases and all-cause and cardiovascular mortality in American women: NHANES 2003–2018
Source: BMC Womens Health. 2024 Apr 5;24:222. doi: 10.1186/s12905-024-03055-6 (PMC10996084; doi:10.1186/s12905-024-03055-6)
Supplement: Supplementary file 2 — Supplementary Material 2 [file 12905_2024_3055_MOESM2_ESM.docx]

**Table 2 Assocaition of reproductive factors with incident CVDs**

**In DM group**

|  |  |  |  |  |
| --- | --- | --- | --- | --- |
| Reproductive Factors | | | Multivariable Model | |
|  | |  | OR (95% CI) | P Value |
| Age at menarche | | |  |  |
| ≤11 | |  | 1.15(0.86-1.55) | 0.342 |
| 12-13 | | ref | | |
| 14-15 | |  | 0.80(0.60-1.05) | 0.104 |
| ≥16 | |  | 1.19(0.69-2.04) | 0.526 |
| Age at menopause | | |  |  |
| ≤44 | |  | 2.06(1.51-2.81) | 0.000 |
| 35-49 | | ref | | |
| 50-54 | |  | 1.02(0.69-1.52) | 0.906 |
| ≥55 | |  | 1.27(0.83-1.95) | 0.265 |
| Reproductive lifespan | | |  |  |
| ≤32 | |  | 1.69(1.13-2.54) | 0.012 |
| 33-35 | | ref | | |
| 36-38 | |  | 0.97(0.58-1.62) | 0.912 |
| 39-41 | |  | 0.64(0.37-1.10) | 0.105 |
| ≥42 | |  | 1.32(0.85-2.06) | 0.214 |
| Maternal age at first live birth | | |  |  |
| ≤19 | |  | 1.17(0.81-1.33) | 0.725 |
| 21-23 | | ref | | |
| 24-26 | |  | 0.84(0.53-1.11) | 0.254 |
| ≥27 | |  | 0.76(0.52-1.08) | 0.321 |
| Maternal age at last live birth | | |  |  |
| ≤26 | |  | 1.40(0.93-2.11) | 0.104 |
| 27-29 | | ref | | |
| 30-34 | |  | 0.96(0.60-1.53) | 0.864 |
| 35-39 | |  | 1.24(0.75-2.06) | 0.396 |
| ≥40 | |  | 0.97(0.39-2.41) | 0.945 |
| Number of pregnancies | | |  |  |
| 1 | |  | 0.80(0.50-1.28) | 0.799 |
| 2 | | ref | | |
| 3 | |  | 0.88(0.57-1.38) | 0.882 |
| 4 | |  | 0.76(0.49-1.17) | 0.757 |
| ≥5 | |  | 1.06(0.73-1.54) | 1.061 |
| Age at menarche | | | 0.94(0.87-1.02) | 0.122 |
| Age at menopause | | | 0.96(0.95-0.97) | 0.000 |
| Reproductive lifespan | | | 0.96(0.95-0.98) | 0.000 |
| Maternal age at first live birth | | | 0.96(0.96-0.97) | 0.001 |
| Maternal age at last live birth | | | 0.99(0.96-1.01) | 0.335 |
| Number of pregnancies | | | 1.05(0.99-1.10) | 0.087 |

**In no-DM group**

| Reproductive Factors | | Multivariable Model | |  |  |
| --- | --- | --- | --- | --- | --- |
|  |  | OR (95% CI) | P Value |  |  |
| Age at menarche | |  |  |  |  |
| ≤11 |  | 1.48(1.12-1.96) | 0.007 |  |  |
| 12-13 | ref | | |  |  |
| 14-15 |  | 1.20(0.94-1.52) | 0.138 |  |  |
| ≥16 |  | 1.300.97-1.74) | 0.076 |  |  |
| Age at menopause | |  |  |  |  |
| ≤44 |  | 1.53(1.21-1.94) | 0.001 |  |  |
| 35-49 | ref | | |  |  |
| 50-54 |  | 0.88(0.65-1.20) | 0.427 |  |  |
| ≥55 |  | 0.79(0.55-1.15) | 0.212 |  |  |
| Reproductive lifespan | |  |  |  |  |
| ≤32 |  | 1.161(1.21-2.14) | 0.001 |  |  |
| 33-35 | ref | | |  |  |
| 36-38 |  | 0.92(0.62-1.37) | 0.693 |  |  |
| 39-41 |  | 1.04(0.67-1.61) | 0.866 |  |  |
| ≥42 |  | 0.87I(0.56-1.33) | 0.510 |  |  |
| Maternal age at first live birth | | |  |  |  |
| ≤19 |  | 1.04(0.81-1.33) | 0.725 |  |  |
| 21-23 | ref | | |  | ref |
| 24-26 |  | 0.74(0.52-1.18) | 0.357 |  |  |
| ≥27 |  | 0.72(0.52-1.08) | 0.321 |  |  |
| Maternal age at last live birth | | |  |  |  |
| ≤26 |  | 0.95(0.71-1.27) | 0.705 |  |  |
| 27-29 | ref | | |  |  |
| 30-34 |  | 0.86(0.62-1.19) | 0.354 |  |  |
| 35-39 |  | 0.93(0.64-1.36) | 0.721 |  |  |
| ≥40 |  | 1.64(0.97-2.77) | 0.066 |  |  |
| Number of pregnancies | |  |  |  |  |
| 1 |  | 1.09(0.75-1.59) | 0.630 |  |  |
| 2 | ref | | |  |  |
| 3 |  | 0.94(0.69-1.30) | 0.716 |  |  |
| 4 |  | 1.34(0.97-1.84) | 0.075 |  |  |
| ≥5 |  | 1.36(1.05-1.76) | 0.021 |  |  |
| Age at menarche | | 0.98(0.92-1.04) | 0.434 |  |  |
| Age at menopause | | 0.97(0.95-0.98) | 0.000 |  |  |
| Reproductive lifespan | | 0.97(0.96-0.98) | 0.000 |  |  |
| Maternal age at first live birth 0.97(0.96-0.98) | | | 0.000 |  |  |
| Maternal age at last live birth | | 1.01(0.99-1.04) | 0.372 |  |  |
| Number of pregnancies | | 1.07(1.02-1.11) | 0.003 |  |  |

**In hypertension group**

| Reproductive Factors | | Multivariable Model | |
| --- | --- | --- | --- |
|  |  | OR (95% CI) | P Value |
| Age at menarche | |  |  |
| ≤11 |  | 1.51(1.14-1.99) | 0.005 |
| 12-13 | ref | | |
| 14-15 |  | 1.20(0.95-1.52) | 0.125 |
| ≥16 |  | 1.26(0.94-1.69) | 0.117 |
| Age at menopause | |  |  |
| ≤44 |  | 1.74(1.42-2.13) | 0.000 |
| 35-49 | ref | | |
| 50-54 |  | 0.90(0.68-1.19) | 0.897 |
| ≥55 |  | 0.86(0.63-1.18) | 0.865 |
| Reproductive lifespan | |  |  |
| ≤32 |  | 1.51(1.19-1.90) | 0.001 |
| 33-35 | ref | | |
| 36-38 |  | 0.82(0.59-1.15) | 0.256 |
| 39-41 |  | 0.80(0.56-1.13) | 0.194 |
| ≥42 |  | 0.84(0.61-1.17) | 0.294 |
| Maternal age at first live birth | | |  |
| ≤19 |  | 114(0.82-1.34) | 0.705 |
| 21-23 | ref | | |
| 24-26 |  | 0.79(0.56-1.19) | 0.426 |
| ≥27 |  | 0.75(0.52-1.09) | 0.327 |
| Maternal age at last live birth | | |  |
| ≤26 |  | 0.01(0.00-0.02) | 0.000 |
| 27-29 | ref | | |
| 30-34 |  | 1.01(0.74-1.38) | 0.939 |
| 35-39 |  | 1.07(0.77-1.50) | 0.686 |
| ≥40 |  | 1.29(0.79-2.10) | 0.308 |
| Number of pregnancies | |  |  |
| 1 |  | 0.94(0.66-1.34) | 0.737 |
| 2 | ref | | |
| 3 |  | 0.88(0.66-1.16) | 0.351 |
| 4 |  | 1.03(0.78-1.36) | 0.842 |
| ≥5 |  | 1.25(0.99-1.57) | 0.060 |
| Age at menarche | | 0.97(0.92-1.03) | 0.318 |
| Age at menopause | | 0.96(0.95-0.97) | 0.000 |
| Reproductive lifespan | | 0.96(0.96-0.97) | 0.000 |
| Maternal age at first live birth 0.98(0.0.97-0.98) | | | 0.000 |
| Maternal age at last live birth | | 101(1.00-1.02) | 0.000 |
| Number of pregnancies | | 1.06(1.02-1.10) | 0.002 |

**In no-hypertension group**

| Reproductive Factors | | Multivariable Model | |
| --- | --- | --- | --- |
|  |  | OR (95% CI) | P Value |
| Age at menarche | |  |  |
| ≤11 |  | 1.53(0.99-2.37) | 0.054 |
| 12-13 | ref | | |
| 14-15 |  | 1.08(0.69-1.68) | 0.743 |
| ≥16 |  | 1.43(0.86-2.39) | 0.168 |
| Age at menopause | |  |  |
| ≤44 |  | 1.53(1.00-2.34) | 0.052 |
| 35-49 | ref | | |
| 50-54 |  | 1.07(0.64-1.78) | 0.788 |
| ≥55 |  | 1.23(0.63-2.42) | 0.545 |
| Reproductive lifespan | |  |  |
| ≤32 |  | 2.39(1.31-4.36) | 0.005 |
| 33-35 | ref | | |
| 36-38 |  | 1.59(0.83-3.06) | 0.161 |
| 39-41 |  | 1.66(0.67-4.08) | 0.267 |
| ≥42 |  | 2.38(1.12-5.05) | 0.024 |
| Maternal age at first live birth | | |  |
| ≤19 |  | 1.08(0.89-1.35) | 0.655 |
| 21-23 | ref | | |
| 24-26 |  | 0.75(0.52-1.19) | 0.425 |
| ≥27 |  | 0.64(0.56-1.08) | 0.326 |
| Maternal age at last live birth | | |  |
| ≤26 |  | 1.28(0.75-2.16) | 0.356 |
| 27-29 | ref | | |
| 30-34 |  | 0.95(0.51-1.77) | 0.868 |
| 35-39 |  | 1.10(0.58-2.10) | 0.758 |
| ≥40 |  | 2.07(0.74-5.81) | 0.164 |
| Number of pregnancies | |  |  |
| 1 |  | 0.94(0.66-1.34) | 0.737 |
| 2 | ref | | |
| 3 |  | 0.88(0.66-1.16) | 0.351 |
| 4 |  | 1.03(0.78-1.36) | 0.842 |
| ≥5 |  | 1.25(0.99-1.57) | 0.060 |
| Age at menarche | | 0.96(0.87-1.06) | 0.414 |
| Age at menopause | | 0.97(0.95-0.99) | 0.011 |
| Reproductive lifespan | | 0.97(0.95-1.00) | 0.025 |
| Maternal age at first live birth 0.97(0.96-0.99) | | | 0.000 |
| Maternal age at last live birth | | 1.00(0.96-1.05) | 0.883 |
| Number of pregnancies | | 1.06(1.02-1.10) | 0.002 |
