# Supplementary material for: Reproductive factors and risk of cardiovascular diseases and all-cause and cardiovascular mortality in American women: NHANES 2003–2018
Source: BMC Womens Health. 2024 Apr 5;24:222. doi: 10.1186/s12905-024-03055-6 (PMC10996084; doi:10.1186/s12905-024-03055-6)
Supplement: Supplementary file 3 — Supplementary Material 3 [file 12905_2024_3055_MOESM3_ESM.docx]

**Table 3 Assocaition of reproductive factors with incident cardic death**

**In DM group**

| Age at menarche | | HR (95% CI) | P Value |  |
| --- | --- | --- | --- | --- |
|  |  |  |  |  |
| ≤11 |  | 0.83(0.63-1.11) | 0.206 |  |
| 12-13 | ref |  |  |  |
| 14-15 |  | 0.84(0.63-1.11) | 0.218 |  |
| ≥16 |  | 1.16(0.82-1.66) | 0.401 |  |
| Age at menopause |  |  |  |  |
| ≤44 |  | 1.18(0.91-1.53) | 0.210 |  |
| 35-49 | ref |  |  |  |
| 50-54 |  | 0.96(0.71-1.30) | 0.797 |  |
| ≥55 |  | 0.91(0.61-1.36) | 0.645 |  |
| Reproductive lifespan |  |  |  |  |
| ≤32 |  | 1.31(0.90-1.92) | 0.158 |  |
| 33-35 | ref |  |  |  |
| 36-38 |  | 1.12(0.72-1.73) | 0.624 |  |
| 39-41 |  | 0.97(0.61-1.54) | 0.887 |  |
| ≥42 |  | 1.09(0.64-1.86) | 0.739 |  |
| Maternal age at first live birth |  |  |  |  |
| ≤19 |  | 1.19(0.67-2.10) | 0.554 |  |
| 21-23 | ref |  |  |  |
| 24-26 |  | 1.04(0.58-1.85) | 0.899 |  |
| ≥27 |  | 1.05(0.55-2.01) | 0.883 |  |
| Maternal age at last live birth |  |  |  |  |
| ≤26 |  | 1.62(1.00-2.61) | 0.050 |  |
| 27-29 | ref |  |  |  |
| 30-34 |  | 1.15(0.72-1.84) | 0.550 |  |
| 35-39 |  | 1.81(1.13-2.90) | 0.014 |  |
| ≥40 |  | 1.16(0.68-1.97) | 0.585 |  |
| Number of fetation |  |  |  |  |
| 1 |  | 1.80(1.19-2.74) | 0.005 |  |
| 2 | ref |  |  |  |
| 3 |  | 1.32(0.92-1.91) | 0.134 |  |
| 4 |  | 1.45(1.02-2.04) | 0.036 |  |
| ≥5 |  | 1.09(0.79-1.50) | 0.595 |  |
| Age at menarche |  | 1.04(0.99-1.09) | 0.147 |  |
| Age at menopause |  | 0.99(0.98-1.00) | 0.062 |  |
| Reproductive lifespan |  | 0.99(0.98-1.00) | 0.049 |  |
| Maternal age at first live birth |  | 0.99(0.96-1.02) | 0.567 |  |
| Maternal age at last live birth |  | 0.99(0.97-1.01) | 0.312 |  |
| Number of pregnancies |  | 0.97(0.92-1.01) | 0.168 |  |

**In no-DM group**

|  |  | HR (95% CI) | P Value |
| --- | --- | --- | --- |
| Age at menarche |  |  |  |
| ≤11 |  | 1.18(0.99-1.39) | 0.061 |
| 12-13 | ref |  |  |
| 14-15 |  | 1.01(0.83-1.22) | 0.938 |
| ≥16 |  | 1.00(0.73-1.35) | 0.975 |
| Age at menopause |  |  |  |
| ≤44 |  | 1.08(0.84-1.39) | 0.553 |
| 35-49 | ref |  |  |
| 50-54 |  | 0.95(0.76-1.19) | 0.650 |
| ≥55 |  | 0.93(0.71-1.23) | 0.634 |
| Reproductive lifespan |  |  |  |
| ≤32 |  | 1.19(0.91-1.56) | 0.207 |
| 33-35 | ref |  |  |
| 36-38 |  | 1.14(0.81-1.59) | 0.451 |
| 39-41 |  | 0.82(0.59-1.15) | 0.254 |
| ≥42 |  | 1.11(0.82-1.49) | 0.506 |
| Maternal age at first live birth |  |  |  |
| ≤19 |  | 0.95(0.63-1.43) | 0.815 |
| 21-23 | ref |  |  |
| 24-26 |  | 0.86(0.62-1.20) | 0.379 |
| ≥27 |  | 1.38(0.95-2.01) | 0.093 |
| Maternal age at last live birth |  |  |  |
| ≤26 |  | 0.77(0.53-1.12) | 0.172 |
| 27-29 | ref |  |  |
| 30-34 |  | 0.93(0.68-1.26) | 0.623 |
| 35-39 |  | 1.14(0.78-1.66) | 0.512 |
| ≥40 |  | 1.30(0.77-2.17) | 0.327 |
| Number of fetation |  |  |  |
| 1 |  | 0.98(0.74-1.31) | 0.905 |
| 2 | ref |  |  |
| 3 |  | 1.06(0.82-1.37) | 0.650 |
| 4 |  | 1.03(0.80-1.32) | 0.820 |
| ≥5 |  | 0.99(0.79-1.25) | 0.960 |
| Age at menarche |  | 0.97(0.92-1.02) | 0.243 |
| Age at menopause |  | 0.99(0.98-1.00) | 0.073 |
| Reproductive lifespan |  | 0.99(0.98-1.00) | 0.165 |
| Maternal age at first live birth |  | 0.99(0.96-1.02) | 0.567 |
| Maternal age at last live birth |  | 1.02(1.00-1.04) | 0.052 |
| Number of pregnancies |  | 0.99(0.95-1.03) | 0.501 |

**In hypertension group**

|  |  | HR (95% CI) | P Value |
| --- | --- | --- | --- |
| Age at menarche |  |  |  |
| ≤11 |  | 1.07(0.91-1.26) | 0.400 |
| 12-13 | ref |  |  |
| 14-15 |  | 0.92(0.76-1.11) | 0.390 |
| ≥16 |  | 1.04(0.81-1.34) | 0.768 |
| Age at menopause |  |  |  |
| ≤44 |  | 1.17(0.96-1.42) | 0.120 |
| 35-49 | ref |  |  |
| 50-54 |  | 0.95(0.77-1.16) | 0.610 |
| ≥55 |  | 1.00(0.76-1.32) | 0.978 |
| Reproductive lifespan |  |  |  |
| ≤32 |  | 1.22(0.94-1.58) | 0.136 |
| 33-35 | ref |  |  |
| 36-38 |  | 1.10(0.82-1.48) | 0.515 |
| 39-41 |  | 0.80(0.61-1.06) | 0.118 |
| ≥42 |  | 1.13(0.83-1.53) | 0.443 |
| Maternal age at first live birth |  |  |  |
| ≤19 |  | 0.96(0.70-1.34) | 0.831 |
| 21-23 | ref |  |  |
| 24-26 |  | 0.84(0.62-1.13) | 0.246 |
| ≥27 |  | 1.42(1.03-1.96) | 0.032 |
| Maternal age at last live birth |  |  |  |
| ≤26 |  | 1.01(0.77-1.33) | 0.946 |
| 27-29 | ref |  |  |
| 30-34 |  | 0.99(0.71-1.39) | 0.951 |
| 35-39 |  | 1.27(0.92-1.76) | 0.148 |
| ≥40 |  | 1.28(0.85-1.93) | 0.245 |
| Number of fetation |  |  |  |
| 1 |  | 1.10(0.84-1.43) | 0.493 |
| 2 | ref |  |  |
| 3 |  | 1.08(0.86-1.35) | 0.529 |
| 4 |  | 1.08(0.84-1.39) | 0.544 |
| ≥5 |  | 0.98(0.80-1.19) | 0.827 |
| Age at menarche |  | 0.97(0.92-1.02) | 0.220 |
| Age at menopause |  | 0.99(0.98-1.00) | 0.006 |
| Reproductive lifespan |  | 0.99(0.98-1.00) | 0.019 |
| Maternal age at first live birth |  | 1.02(0.99-1.05) | 0.154 |
| Maternal age at last live birth |  | 1.01(1.00-1.03) | 0.170 |
| Number of pregnancies |  | 0.98(0.95-1.01) | 0.205 |

**In no-hypertension group**

|  |  | HR (95% CI) | P Value |
| --- | --- | --- | --- |
| Age at menarche |  |  |  |
| ≤11 |  | 0.98(0.72-1.33) | 0.879 |
| 12-13 | ref | | |
| 14-15 |  | 1.06(0.79-1.40) | 0.713 |
| ≥16 |  | 1.05(0.65-1.71) | 0.835 |
| Age at menopause |  |  |  |
| ≤44 |  | 0.98(0.67-1.42) | 0.907 |
| 35-49 | ref | | |
| 50-54 |  | 1.01(0.68-1.51) | 0.952 |
| ≥55 |  | 0.63(0.36-1.12) | 0.118 |
| Reproductive lifespan |  |  |  |
| ≤32 |  | 1.38(0.89-2.13) | 0.148 |
| 33-35 | ref | | |
| 36-38 |  | 1.33(0.78-2.25) | 0.298 |
| 39-41 |  | 1.23(0.69-2.21) | 0.485 |
| ≥42 |  | 0.95(0.54-1.67) | 0.851 |
| Maternal age at first live birth |  |  |  |
| ≤19 |  | 1.23(0.54-2.83) | 0.620 |
| 21-23 | ref | | |
| 24-26 |  | 1.21(0.52-2.81) | 0.652 |
| ≥27 |  | 0.78(0.26-2.31) | 0.652 |
| Maternal age at last live birth |  |  |  |
| ≤26 |  | 0.92(0.49-1.70) | 0.781 |
| 27-29 | ref | | |
| 30-34 |  | 0.84(0.48-1.50) | 0.563 |
| 35-39 |  | 1.39(0.69-2.80) | 0.356 |
| ≥40 |  | 0.92(0.37-2.30) | 0.859 |
| Number of fetation |  |  |  |
| 1 |  | 1.62(0.93-2.82) | 0.087 |
| 2 | ref | | |
| 3 |  | 1.21(0.78-1.88) | 0.394 |
| 4 |  | 1.40(0.88-2.23) | 0.155 |
| ≥5 |  | 1.19(0.83-1.72) | 0.352 |
| Age at menarche |  | 1.03(0.96-1.11) | 0.366 |
| Age at menopause |  | 0.98(0.97-0.99) | 0.001 |
| Reproductive lifespan |  | 0.99(0.97-1.01) | 0.409 |
| Maternal age at first live birth |  | 1.02(0.96-1.07) | 0.600 |
| Maternal age at last live birth |  | 1.00(0.96-1.04) | 0.879 |
| Number of pregnancies |  | 0.98(0.95-1.01) | 0.205 |
