# Supplementary material for: Reproductive factors and risk of cardiovascular diseases and all-cause and cardiovascular mortality in American women: NHANES 2003–2018
Source: BMC Womens Health. 2024 Apr 5;24:222. doi: 10.1186/s12905-024-03055-6 (PMC10996084; doi:10.1186/s12905-024-03055-6)
Supplement: Supplementary file 4 — Supplementary Material 4 [file 12905_2024_3055_MOESM4_ESM.docx]

Table 4 Assocaition of reproductive factors with incident death

**In DM group**

| Reproductive Factors | | Multivariable Model | |
| --- | --- | --- | --- |
|  |  | HR (95% CI) | P Value |
| Age at menarche | |  |  |
| ≤11 |  | 0.82(0.52-1.28) | 0.381 |
| 12-13 | ref | | |
| 14-15 |  | 0.72(0.51-1.02) | 0.066 |
| ≥16 |  | 1.51(0.92-2.50) | 0.105 |
| Age at menopause | |  |  |
| ≤44 |  | 1.30(0.79-2.14) | 0.294 |
| 35-49 | ref | | |
| 50-54 |  | 0.79(0.41-1.53) | 0.491 |
| ≥55 |  | 0.80(0.41-1.57) | 0.513 |
| Reproductive lifespan | |  |  |
| ≤32 |  | 2.38(1.36-4.18) | 0.003 |
| 33-35 | ref | | |
| 36-38 |  | 1.53(0.75-3.14) | 0.246 |
| 39-41 |  | 1.21(0.56-2.62) | 0.632 |
| ≥42 |  | 1.17(0.57-2.38) | 0.666 |
| Maternal age at first live birth | | |  |
| ≤19 |  | 1.10(0.47-2.57) | 0.822 |
| 21-23 | ref | | |
| 24-26 |  | 0.65(0.24-1.77) | 0.400 |
| ≥27 |  | 1.27(0.43-3.74) | 0.662 |
| Maternal age at last live birth | | |  |
| ≤26 |  | 1.38(0.76-2.49) | 0.293 |
| 27-29 | ref | | |
| 30-34 |  | 0.90(0.41-1.98) | 0.789 |
| 35-39 |  | 0.87(0.45-1.70) | 0.690 |
| ≥40 |  | 0.58(0.25-1.36) | 0.212 |
| Number of fetation | |  |  |
| 1 |  | 2.32(1.16-4.65) | 0.018 |
| 2 | ref | | |
| 3 |  | 1.57(0.84-2.96) | 0.160 |
| 4 |  | 1.58(0.86-2.89) | 0.137 |
| ≥5 |  | 0.94(0.57-1.56) | 0.819 |
| Age at menarche | | 1.07(0.98-1.17) | 0.143 |
| Age at menopause | | 0.98(0.96-0.99) | 0.012 |
| Reproductive lifespan | | 0.97(0.96-0.99) | 0.003 |
| Maternal age at first live birth | | 0.97(0.90-1.04) | 0.399 |
| Maternal age at last live birth | | 0.94(0.90-0.99) | 0.013 |
| Number of pregnancies | | 0.88(0.81-0.95) | 0.002 |
|  |  |  |  |

**In no-DM group**

| Reproductive Factors | | Multivariable Model | |
| --- | --- | --- | --- |
|  |  | HR (95% CI) | P Value |
| Age at menarche | |  |  |
| ≤11 |  | 1.11(0.73-1.70) | 0.613 |
| 12-13 | ref |  |  |
| 14-15 |  | 1.21(0.82-1.80) | 0.334 |
| ≥16 |  | 0.92(0.56-1.51) | 0.749 |
| Age at menopause | |  |  |
| ≤44 |  | 1.14(0.67-1.95) | 0.635 |
| 35-49 | ref |  |  |
| 50-54 |  | 1.03(0.67-1.57) | 0.902 |
| ≥55 |  | 0.84(0.49-1.44) | 0.519 |
| Reproductive lifespan | |  |  |
| ≤32 |  | 1.32(0.76-2.29) | 0.325 |
| 33-35 | ref |  |  |
| 36-38 |  | 1.09(0.67-1.77) | 0.729 |
| 39-41 |  | 1.05(0.59-1.87) | 0.865 |
| ≥42 |  | 0.84(0.44-1.61) | 0.607 |
| Maternal age at first live birth | | |  |
| ≤19 |  | 1.10(0.47-2.57) | 0.822 |
| 21-23 | ref |  |  |
| 24-26 |  | 0.65(0.24-1.77) | 0.400 |
| ≥27 |  | 1.27(0.43-3.74) | 0.662 |
| Maternal age at last live birth | | |  |
| ≤26 |  | 0.73(0.33-1.60) | 0.426 |
| 27-29 | ref |  |  |
| 30-34 |  | 1.16(0.53-2.57) | 0.709 |
| 35-39 |  | 1.13(0.53-2.42) | 0.756 |
| ≥40 |  | 1.15(0.42-3.16) | 0.787 |
| Number of fetation | |  |  |
| 1 |  | 1.34(0.71-2.52) | 0.372 |
| 2 | ref |  |  |
| 3 |  | 1.04(0.66-1.64) | 0.864 |
| 4 |  | 1.28(0.77-2.12) | 0.339 |
| ≥5 |  | 1.24(0.76-2.03) | 0.387 |
| Age at menarche | | 1.01(0.92-1.11) | 0.834 |
| Age at menopause | | 0.98(0.97-1.00) | 0.115 |
| Reproductive lifespan | | 0.98(0.96-1.00) | 0.091 |
| Maternal age at first live birth | | 0.97(0.90-1.04) | 0.399 |
| Maternal age at last live birth | | 1.02(0.99-1.05) | 0.193 |
| Number of pregnancies | | 1.02(0.96-1.10) | 0.510 |

**In hypertension group**

| Reproductive Factors | | Multivariable Model | |
| --- | --- | --- | --- |
|  |  | HR (95% CI) | P Value |
| Age at menarche | |  |  |
| ≤11 |  | 1.13(0.74-1.71) | 0.581 |
| 12-13 | ref | | |
| 14-15 |  | 1.22(0.82-1.80) | 0.333 |
| ≥16 |  | 0.92(0.56-1.51) | 0.736 |
| Age at menopause | |  |  |
| ≤44 |  | 1.23(0.87-1.76) | 0.246 |
| 35-49 | ref | | |
| 50-54 |  | 0.91(0.65-1.27) | 0.560 |
| ≥55 |  | 0.84(0.53-1.31) | 0.434 |
| Reproductive lifespan | |  |  |
| ≤32 |  | 1.51(0.98-2.32) | 0.062 |
| 33-35 | ref | | |
| 36-38 |  | 1.04(0.68-1.59) | 0.845 |
| 39-41 |  | 0.99(0.65-1.51) | 0.963 |
| ≥42 |  | 0.83(0.48-1.42) | 0.494 |
| Maternal age at first live birth | | |  |
| ≤19 |  | 1.40(0.74-2.65) | 0.305 |
| 21-23 | ref | | |
| 24-26 |  | 0.96(0.52-1.79) | 0.904 |
| ≥27 |  | 1.59(0.80-3.16) | 0.185 |
| Maternal age at last live birth | | |  |
| ≤26 |  | 1.12(0.65-1.91) | 0.682 |
| 27-29 | ref | | |
| 30-34 |  | 1.06(0.57-2.00) | 0.849 |
| 35-39 |  | 1.18(0.65-2.12) | 0.589 |
| ≥40 |  | 1.07(0.58-1.99) | 0.826 |
| Number of fetation | |  |  |
| 1 |  | 1.62(0.93-2.82) | 0.087 |
| 2 | ref | | |
| 3 |  | 1.21(0.78-1.88) | 0.394 |
| 4 |  | 1.40(0.88-2.23) | 0.155 |
| ≥5 |  | 1.19(0.83-1.72) | 0.352 |
| Age at menarche | | 1.01(0.92-1.11) | 0.893 |
| Age at menopause | | 0.98(0.97-0.99) | 0.001 |
| Reproductive lifespan | | 0.98(0.97-0.99) | 0.000 |
| Maternal age at first live birth | | 1.01(0.96-1.07) | 0.643 |
| Maternal age at last live birth | | 0.99(0.96-1.03) | 0.588 |
| Number of pregnancies | | 0.97(0.92-1.03) | 0.287 |

**In no-hypertension group**

| Reproductive Factors | | Multivariable Model | |  |  |  |  |  |  |
| --- | --- | --- | --- | --- | --- | --- | --- | --- | --- |
|  |  | HR (95% CI) | P Value |  |  |  |  |  |  |
| Age at menarche | |  |  |  |  |  |  |  |  |
| ≤11 |  | 0.98(0.72-1.33) | 0.879 |  |  |  |  |  |  |
| 12-13 | ref | | |  |  |  |  |  |  |
| 14-15 |  | 1.06(0.79-1.40) | 0.713 |  |  |  |  |  |  |
| ≥16 |  | 1.05(0.65-1.71) | 0.835 |  |  |  |  |  |  |
| Age at menopause | |  |  |  |  |  |  |  |  |
| ≤44 |  | 0.98(0.67-1.42) | 0.907 |  |  |  |  |  |  |
| 35-49 | ref | | |  |  |  |  |  |  |
| 50-54 |  | 1.01(0.68-1.51) | 0.952 |  |  |  |  |  |  |
| ≥55 |  | 0.63(0.36-1.12) | 0.118 |  |  |  |  |  |  |
| Reproductive lifespan | |  |  |  |  |  |  |  |  |
| ≤32 |  | 1.51(0.98-2.32) | 0.062 |  |  |  |  |  |  |
| 33-35 | ref | | |  | | | ref | | |
| 36-38 |  | 1.04(0.68-1.59) | 0.845 |  |  |  |  |  |  |
| 39-41 |  | 0.99(0.65-1.51) | 0.963 |  |  |  |  |  |  |
| ≥42 |  | 0.83(0.48-1.42) | 0.494 |  |  |  |  |  |  |
| Maternal age at first live birth | | |  |  |  |  |  |  |  |
| ≤19 |  | 1.45(0.76-2.68) | 0.305 |  |  |  |  |  |  |
| 21-23 | ref | | |  | | ref | | |  |
| 24-26 |  | 0.97(0.53-1.89) | 0.925 |  |  |  |  |  |  |
| ≥27 |  | 1.23(0.70-3.17) | 0.189 |  |  |  |  |  |  |
| Maternal age at last live birth | | |  |  |  |  |  |  |  |
| ≤26 |  | 1.16(0.67-1.95) | 0.724 |  |  |  |  |  |  |
| 27-29 | ref | | |  | ref | | |  |  |
| 30-34 |  | 1.08(0.58-2.01) | 0.849 |  |  |  |  |  |  |
| 35-39 |  | 1.19(0.66-2.17) | 0.615 |  |  |  |  |  |  |
| ≥40 |  | 1.08(0.59-1.99) | 0.828 |  |  |  |  |  |  |
| Number of fetation | |  |  |  |  |  |  |  |  |
| 1 |  | 1.62(0.93-2.82) | 0.087 |  |  |  |  |  |  |
| 2 | ref | | |  |  |  |  |  |  |
| 3 |  | 1.21(0.78-1.88) | 0.394 |  |  |  |  |  |  |
| 4 |  | 1.40(0.88-2.23) | 0.155 |  |  |  |  |  |  |
| ≥5 |  | 1.19(0.83-1.72) | 0.352 |  |  |  |  |  |  |
| Age at menarche | | 1.03(0.96-1.11) | 0.366 |  |  |  |  |  |  |
| Age at menopause | |  |  |  |  |  |  |  |  |
| Reproductive lifespan | | 0.99(0.97-1.01) | 0.409 |  |  |  |  |  |  |
| Maternal age at first live birth 1.02(0.95-1.09) | | | 0.756 |  |  |  |  |  |  |
| Maternal age at last live birth 0.97(0.95-1.04) | | | 0.526 |  |  |  |  |  |  |
| Number of pregnancies | | 0.97(0.92-1.03) | 0.287 |  |  |  |  |  |  |
